# Obligate Biotroph Pathogens of the Genus *Albugo* Are Better Adapted to Active Host Defense Compared to Niche Competitors

**DOI:** 10.3389/fpls.2016.00820

**Published:** 2016-06-20

**Authors:** Jonas Ruhe, Matthew T. Agler, Aleksandra Placzek, Katharina Kramer, Iris Finkemeier, Eric M. Kemen

**Affiliations:** ^1^Max Planck Institute for Plant Breeding ResearchCologne, Germany; ^2^Institute of Plant Biology and Biotechnology, University of MuensterMünster, Germany

**Keywords:** biotrophy, *Albugo*, apoplast, proteomics, field studies, microbe–microbe interactions, systemic acquired resistance, immunity

## Abstract

Recent research suggested that plants behave differently under combined versus single abiotic and biotic stress conditions in controlled environments. While this work has provided a glimpse into how plants might behave under complex natural conditions, it also highlights the need for field experiments using established model systems. In nature, diverse microbes colonize the phyllosphere of *Arabidopsis thaliana*, including the obligate biotroph oomycete genus *Albugo*, causal agent of the common disease white rust. Biotrophic, as well as hemibiotrophic plant pathogens are characterized by efficient suppression of host defense responses. Lab experiments have even shown that *Albugo* sp. can suppress non-host resistance, thereby enabling otherwise avirulent pathogen growth. We asked how a pathogen that is vitally dependent on a living host can compete in nature for limited niche space while paradoxically enabling colonization of its host plant for competitors? To address this question, we used a proteomics approach to identify differences and similarities between lab and field samples of *Albugo* sp.-infected and -uninfected *A. thaliana* plants. We could identify highly similar apoplastic proteomic profiles in both infected and uninfected plants. In wild plants, however, a broad range of defense-related proteins were detected in the apoplast regardless of infection status, while no or low levels of defense-related proteins were detected in lab samples. These results indicate that *Albugo* sp. do not strongly affect immune responses and leave distinct branches of the immune signaling network intact. To validate our findings and to get mechanistic insights, we tested a panel of *A. thaliana* mutant plants with induced or compromised immunity for susceptibility to different biotrophic pathogens. Our findings suggest that the biotroph pathogen *Albugo* selectively interferes with host defense under different environmental and competitive pressures to maintain its ecological niche dominance. Adaptation to host immune responses while maintaining a partially active host immunity seems advantageous against competitors. We suggest a model for future research that considers not only host–microbe but in addition microbe–microbe and microbe–host environment factors.

## Introduction

In the wild, plants are exposed simultaneously to a variety of environmental stresses. Plant responses to biotic stress caused by microbes or abiotic stress like heat, starvation, or drought are largely distinct, but partly overlapping (Richards et al., 2012). In the laboratory, specific conditions are usually tested in isolation to study how plants react to stress on a molecular level. Ultimately, researchers would like to apply this knowledge to crop plants under field conditions and for this it is important to elucidate how laboratory-based knowledge transfers into nature.

*Arabidopsis thaliana* is the best-studied species of flowering plant (Koornneef and Meinke, 2010), but knowledge about how it behaves in the wild on a molecular level is still limited. This is because abiotic and biotic stimuli constantly fluctuate during wild plant growth, limiting replicability of field experiments. Under controlled conditions several studies examined how plants behave in response to single factors like salt and osmotic stress (Kreps et al., 2002), temperature change (Fowler and Thomashow, 2002; Kreps et al., 2002; Rizhsky et al., 2004) and drought (Rabello et al., 2008), starvation (Palenchar et al., 2004; Lian et al., 2006), or biotic stresses (Schenk et al., 2000; De Vos et al., 2005; Amrine et al., 2015; Lewis et al., 2015). Responses apparently interact, however, since gene expression analysis demonstrated that single stress responses cannot sufficiently predict changes after combined stresses (Mittler, 2006; Rasmussen et al., 2013). Therefore, to understand, for example, biotic stress responses, lab experiments with single stresses in isolation should be compared to the same stress under natural conditions.

The first studies on plant behavior under natural conditions showed that the transcriptome is mostly affected by circadian rhythm, environmental stimuli, and plant age (Nagano et al., 2012; Richards et al., 2012). Many gene clusters co-expressed under natural conditions are enriched in loci responsive to (a-)biotic stimuli, which suggests that stress-responsive genes are deployed during the whole life cycle of *A. thaliana* (Richards et al., 2012). It is not clear yet how these transcription results translate into protein abundances in the field.

In the field, white blister rust, caused by the obligate biotroph oomycete genus *Albugo*, is one of the most widespread diseases of Brassicaceae (Ploch and Thines, 2011). *Albugo* sp. enter via stomata, form intercellular hyphae, penetrate the plant cell wall and invaginate the plant plasma membrane with haustoria in order to take up plant nutrients and release effector proteins into host cells (Kemen et al., 2005; Rafiqi et al., 2010; Spanu and Kämper, 2010; Kemen and Jones, 2012). To complete their whole lifecycle on the living host plant, obligate biotroph pathogens must be highly adapted to the host. Research on the effector complement of hemibiotroph and biotroph pathogens has already provided insights into how efficiently these pathogens interact with their respective host (De Wit et al., 2009; Caillaud et al., 2013; Asai et al., 2014; Irieda et al., 2014). For example, the biotrophic downy mildew pathogen *Hyaloperonospora arabidopsidis* (*Hpa*), which has an overlapping host range with *Albugo* sp., and suppresses host responsiveness to salicylic acid (SA) in infected cells (Asai et al., 2014). It is known that hemi-biotrophic and necrotrophic pathogens trigger host secretion to the apoplast of defense-related proteins including pathogenesis-related (PR) proteins (Floerl et al., 2008, 2012; Ali et al., 2014; Kim et al., 2014). However, the influence of obligate biotroph pathogens on the *A. thaliana* apoplastic secretome is still unknown.

Host plant colonization by obligate biotrophs of the genus *Albugo* is associated with suppression of non-host resistance (NHR; Cooper et al., 2008). Specifically, *Albugo* sp. suppressed the “runaway cell death” phenotype, allowing a formerly avirulent downy mildew species to infect (Cooper et al., 2008). Assuming this phenomenon would extend to other non-host pathogens, *Albugo* sp. could thereby influence the microbial community composition of the host. We previously used network modeling of microbial community structures in the phyllosphere to show that the community composition is not only affected by abiotic factors and host genotype, but also by microbial hubs, including *Albugo* (Agler et al., 2016). Microbial hubs are taxa that are highly interconnected in a microbial community network and have significant effects on the community structure (Agler et al., 2016). It is still unclear whether hubs affect microbial colonization via direct microbe–microbe interactions or if they depend on host-mediated defense responses like suppression of NHR and effector-triggered immunity (ETI).

We investigated how *Albugo*, which is vitally dependent on a living host, can paradoxically compete in nature for a limited niche while in effect breaking host resistance to colonization. This was done by performing shotgun proteomics on leaf apoplastic fluid samples of *Albugo* sp.-infected and -uninfected *A. thaliana*. We examined plant samples from two different wild sites and a common garden experiment to compare the results with experiments performed under controlled laboratory conditions. Our results conclusively show that both wild-grown and lab-grown *A. thaliana* supports extensive *Albugo* sp. colonization, but the secretomes between wild and lab plants were significantly different. Regardless of *Albugo* sp. infection status, wild plants showed a broad spectrum of defense-related proteins at high abundances and lab-grown plants did not. We hypothesized that the activated immune system in wild plants leads to high hormone levels of, e.g., SA or abscisic acid (ABA). We used *A. thaliana* hormone mutants to mimic variable hormone levels in the field and found that *Albugo laibachii* strains are less affected in their infection rate than the natural competitor *Hpa*. Thus, our findings reveal how the biotroph pathogen *Albugo* only selectively interferes with host defense under different environmental conditions to maintain its ecological niche. Adaptation to host immune responses while maintaining partially active immunity seems advantageous against competitors.

## Materials and Methods

### Plant Growth Conditions

*Arabidopsis thaliana* seeds were stratified on moist soil for 7 days at 4°C in darkness, before transfer into growth chambers with short day conditions (10 h light, 14 h darkness, 23/20°C, constant 60% humidity). Plants were grown for 6 weeks prior to infection, except ABA mutants which were grown for 4 weeks. For apoplastic fluid proteomics experiments in the lab (see Extraction of Apoplastic Fluid from *A. thaliana* Leaves), *A. thaliana* Ws-0 was used. Since all mutants used for qPCR experiments (see DNA Extraction and Oomycete Growth Quantification via qPCR) were in Col-0 background, wild type Col-0 was used as a control for infections in these experiments.

### *Arabidopsis thaliana* Infections with Oomycete Pathogen Isolates

Spore solutions were prepared by washing spores from *Albugo candida* Nc2 or *A. laibachii* Nc14 or MPI1 infected leaf material with tap water and held on ice for 1 h. One milliliter spore solution was sprayed per plant (16 × 10^4^ spores/ml) on 6-week-old *A. thaliana* using airbrush guns (Conrad Electronics GmbH). Plants were kept overnight in a dark cold room to promote spore germination, then were further grown in cabinets (Sanyo Inc.) with a dark–light cycle of 10 h light at 22°C and 14 h darkness at 16°C.

Infections with *Hpa* Noco2 were done similarly by washing infected leaves to make a conidiospore solution (4 × 10^4^ spores/ml), then spraying on *A. thaliana* plants (Stuttmann et al., 2011).

### Sampling of Wild *A. thaliana* Plants

Wild *A. thaliana* plants were sampled in Pulheim (Pul, 50°59′08.5′′N 6°49′35.4′′E) and Geyen (Gey, 50°58′43.9′′N 6°47′35.8′′E). Leaf material was pooled from several plants with white rust infection symptoms or asymptomatic plants at each site to reach appropriate amounts (≥2 g) for apoplastic fluid extractions. While sampling asymptomatic plants, care was taken to use only leaves where the whole plant did not show any white rust sporulation. Sampling was done in spring and fall 2013 (only fall at Gey) and in spring 2014. A third (fourth) replicate was also taken from Gey (Pul) in spring 2015, but liquid chromatography tandem mass spectrometry (LC-MS/MS) analysis (see below) was performed with an updated workflow combining in-solution digestion and analysis on a Q Exactive Plus. This increased sensitivity, coverage and resolution; the absolute values are therefore not comparable with samples analyzed on the LTQ Velos after in-gel digestion.

Growth of plants in the common garden experiment at the Max-Planck-Institute, Cologne, Germany, was described in Agler et al. (2016). *A. thaliana* Ws-0 and Sf-2, which is an *A. thaliana* ecotype resistant to *Albugo* infections, were planted and sampling was performed exactly as described for the wild sites.

### Extraction of Apoplastic Fluid from *A. thaliana* Leaves

For laboratory experiments, ∼7 g of leaves were harvested from uninfected mock (H_2_O sprayed) or *Albugo* sp.-infected (10 dpi) plants and immersed in 180 mM 2-(*N*-morpholino)ethanesulfonic acid (MES) (Duchefa Biochemie; pH 5.5) infiltration buffer. Leaves sampled from natural sites or the common garden experiment (2–9 g) were washed twice with ddH_2_O to reduce dirt that was sticking to the leaves prior to immersion in infiltration buffer. A vacuum was applied with three cycles of 2:30 min followed by slow release over the course of 10 min to infiltrate the leaves homogenously. The leaves were blot-dried with tissue and placed in a 50 ml falcon tube with holes in the bottom in a centrifugation bottle. Apoplastic fluid was collected via centrifugation (Thermo Scientific) at 4°C and 900 ×*g* for 10 min and was then sterile filtered (0.22 μm syringe filter, Spectrum Labs). Proteins were precipitated via chloroform/methanol precipitation (Wessel and Flügge, 1984). Pellets were dissolved in Laemmli sample buffer (Laemmli, 1970) for in-gel digestion and stored at -20°C until LC-MS/MS analysis.

### LC–MS/MS Analysis

For samples analyzed with the LTQ Velos machine (lab and wild samples replicates 1–3), about 20 μg of apoplast protein extract per sample was separated at 125 V on a linear 12% Tris-Glycine sodium dodecyl sulfate-polyacrylamide gel electrophoresis (SDS-PAGE) for approximately 2 h. Gels were stained with PageBlue protein staining solution (Fermentas). Each gel lane was dissected into 30–32 slices. Gel slices were destained, reduced, alkylated, and trypsinated by a Proteineer dp robot (Bruker Daltonics). Peptides were separated on a Thermo/Proxeon Easy nLC II in a two-column configuration (precolumn 3 cm × 100 μm, 5 μm C18AQ medium, analytical column 10 cm × 75 μm, 3 μm C18AQ) coupled to a LTQ-Velos ion trap (Thermo Scientific). Peptides were eluted over a segmented linear 130 min gradient running from 5 to 95% acetonitrile (ACN)/H_2_O with 0.5% formic acid (FA) at a flow-rate of 300 nl/min. Survey full-scan mass spectra were acquired in a mass range from 400 to 1600 *m/z*. MS/MS spectra were acquired with collision-induced dissociation (CID) at 35 eV on multiply charged precursor ions using a Top10 method with active exclusion for 60 s in a window from 0.2 Da below to 1.5 Da above the precursor mass. The ion selection threshold was set to 500 counts for MS2, the activation Q was set to 0.25 and the activation time to 10 ms. The resulting RAW files were converted to MGF format using Proteome discoverer 1.4.0288 (Thermo Scientific).

For samples analyzed on the Q Exactive Plus machine (lab samples, wild sample replicate 4 and common garden experiment samples), 5–20 μg apoplast protein extract per sample were digested in solution. The protein pellet was dissolved in 8 M urea, 0.1 M Tris–HCl pH 8.0, 1 mM CaCl_2_. Cysteins were reduced by adding dithiothreitol (DTT) to a final concentration of 5 mM and incubation for 30 min. Subsequently, alkylation was performed by adding chloroacetamide to a final concentration of 14 mM and incubation for 30 min. The reaction was quenched by addition of DTT. Urea concentration was adjusted to 2 M by dilution with 0.1 M Tris–HCl pH 8.0, 1 mM CaCl_2_. Trypsin digestion (1:100 enzyme-to-protein ratio) was performed over night at 37°C and stopped by addition of 1% FA. Peptides were desalted with StageTips (Empore C18, 3 M) as described in Rappsilber et al. (2007). Dried peptides were redissolved in 2% ACN, 0.1% trifluoroacetic acid for analysis and adjusted to a final concentration of 0.18 μg/μl. Samples were analyzed using an EASY-nLC 1000 (Thermo Fisher) coupled to a Q Exactive Plus mass spectrometer (Thermo Fisher). Peptides were separated on 16 cm frit-less silica emitters (New Objective, 0.75 μm inner diameter), packed in-house with reversed-phase ReproSil-Pur C18 AQ 3 μm resin (Dr. Maisch). Peptides (1 μg) were loaded on the column and eluted for 120 min using a segmented linear gradient of 0–95% solvent B (solvent A 5% ACN, 0.5% FA; solvent B 100% ACN, 0.5% FA) at a flow-rate of 250 nl/min. Mass spectra were acquired in data-dependent acquisition mode with a Top15 method. MS spectra were acquired in the Orbitrap analyzer with a mass range of 300–1750 *m/z* at a resolution of 70,000 FWHM (full width at half maximum) and a target value of 3 × 10^6^ ions. Precursors were selected with an isolation window of 1.3 *m/z*. High-energy collisional dissociation fragmentation was performed at a normalized collision energy of 25. MS/MS spectra were acquired with a target value of 10^5^ ions at a resolution of 17,500 FWHM and a fixed first mass of *m/z* 100. Peptides with a charge of +1, greater than 6, or with unassigned charge state were excluded from fragmentation for MS2, dynamic exclusion for 30 s prevented repeated selection of precursors.

### MS Data Processing

MS/MS spectra were searched against the *A. thaliana* proteome (TAIR10) and against the *A. laibachii* Nc14/*A. candida* Nc2 (Kemen et al., 2011; McMullan et al., 2015) proteomes. A database containing 248 common contaminants and reverse “decoy” sequences were included. Trypsin specificity was required and a maximum of two missed cleavages allowed. Carbamidomethylation of cysteine residues was set as fixed modification and oxidation of methionine as variable modification. LTQ Velos data was searched using X! Tandem (version 2013.02.01.1; Craig and Beavis, 2004). A fragment monoisotopic mass error of 0.4 Da was permitted with a parent monoisotopic mass error of ±0.3 Da. Protein identifications were validated via the trans-proteomic-pipeline (TPP version 4.6.2; Deutsch et al., 2010), using default settings. For refinement and quantification of protein identifications APEX spectral counting (version 1.1.0) was used (Braisted et al., 2008). The APEX abundances of observed proteins were calculated using the results from PeptideProphet and ProteinProphet analyses (part of TPP) and a false discovery rate (FDR) of 1%. For further analyses proteins were filtered for secreted proteins, based on TargetP 1.1 predictions (Emanuelsson et al., 2000) (Supplementary Material: Tables S3–S7).

Q Exactive data were processed using MaxQuant software (version 1.5.2.8^1^; Cox and Mann, 2008) with label-free quantification (LFQ) and iBAQ enabled (Cox et al., 2014). Minimal peptide length was set to seven amino acids. Peptide-spectrum-matches and proteins were retained if they were below a FDR of 1%. Subsequent quantitative statistical analyses were performed in Perseus (version 1.5.2.6; Cox and Mann, 2012). LFQ intensities were log2 transformed. iBAQ values were used to filter for the 100 most abundant proteins, which were further analyzed based on their LFQ values (Supplementary Material: Table S8).

Due to different mass spectrometers and sample preparations a direct comparison based on protein abundances is not possible for samples analyzed with different setups.

### Statistical Analyses of Proteomics Samples

To evaluate how the composition of proteomic samples varied between different treatments and sampling sites, we used redundancy analyses (RDA) in the R-package Vegan 2.2-1 (Oksanen et al., 2015). We first built an unconstrained model with the command RDA of all proteins in the sample with their respective APEX or LFQ abundance value. This model was then constrained by the factors location, infection status, or sample type (treatment). For visualization, we plotted the first constrained axes (if the factor was only two levels, only one constrained and one unconstrained axis are generated) and calculated the total variation of protein composition that was correlated to the constraining variables. To test if the constraining was significant (*p* < 0.05), we used the ANOVA function built into Vegan, which uses random permutations of factor classes followed by Tukey honestly significant difference (HSD) in R 3.0.2 (R Core Team, 2013). Ellipses of confidence intervals were plotted to help visualizing significant correlations. The built-in ordiellipse function was used to calculate the 95% confidence limits based on standard error.

RDA with all of the Q Exactive Plus-measured samples, constrained for the factor “treatments,” indicated that five samples strongly diverged from their respective replicates (Supplementary Figure S1A). Due to technical reasons, these samples had abnormal MS/MS identification rates and showed low correlation to all other samples (Supplementary Figure S1B). For further analyses, these samples were excluded.

To determine if asymptomatic wild-grown *A. thaliana* plants were free of *Albugo* growth, the identified MS/MS spectra of these samples were searched against the *A. laibachii* Nc14 proteome and *A. thaliana* proteome and the ratio of *Albugo* proteins/*A. thaliana* proteins was compared with the ratios in *Albugo*-infected samples (Supplemenatry Tables S1 and S2).

### DNA Extraction and Oomycete Growth Quantification via qPCR

Plants from the mutant infection assay were harvested at 10 dpi (*A. laibachii/A. candida* infections or mock) or at 5 dpi (*Hpa* Noco2 infections or mock) and were immediately frozen in tubes in liquid nitrogen. Three adult plants (or five seedlings) were pooled and ground to powder using a liquid nitrogen-cooled mortar and pestle and DNA was extracted following a phenol/chloroform-extraction protocol (McKinney et al., 1995). In short, ground powder was taken, added to extraction buffer (50 mM Tris pH 8.0, 200 mM NaCl, 0.2 mM ethylenediaminetetraacetic acid (EDTA), 0.5% SDS, 0.1 mg/ml proteinase K (Sigma–Aldrich) and incubated at 37°C for 30 min. One volume phenol was added, centrifuged and the top aqueous layer recovered and was mixed with 1 volume chloroform/isoamyl alcohol (24:1; Sigma-Aldrich). After centrifugation, the top aqueous layer was recovered and mixed with 3 M sodium acetate and two volumes pure ethanol to precipitate the nucleic acids. DNA was pelleted by centrifugation and washed twice with 70% ethanol. It was resuspended in 50 μl nuclease free water (NFW) and used for qPCR. DNA concentrations were determined via NanoDrop (Thermo Scientific) and diluted to 1 ng/μl. One qPCR reaction contained 7.5 μl SsoAdvanced universal SYBR Green supermix (Bio-Rad), 0.3 μl of each primer (10 mM), 1.9 μl NFW and 5 μl DNA. Samples were measured in triplicates in a CFX Connect real-time PCR detection system (Bio-Rad) using the following program: (1) 95°C, 2 min; (2) (95°C, 20 s, then 56°C, 20 s, then 72°C, 30 s) ×40, 72°C, 5 min followed by a temperature gradient from 65 to 95°C. To quantify the amount of oomycete DNA per plant sample two standard genes were used (*A. thaliana* EF1-α: 5′-AAGGAGGCTGCTGAGATGAA-3′, 5′-TGGTGGTCTCGAACTTCCAG-3′; Oomycete internal transcribed spacer (ITS) 5.8s: 5′-ACTTTCAGCAGTGGATGTCTA-3′, 5′-GATGACTCACTGAATTCTGCA-3′). The amount of oomycete DNA was normalized to the respective plant DNA content and infected plant mutants were normalized to infected Col-0 wild type plants via calculating the ΔΔ*C*q.

### Amplicon Sequencing of Microbial Communities

DNA extraction, amplicon library preparation, and sequencing were performed as previously described (Agler et al., 2016). In short, DNA was extracted with bead beating and SDS/lysozyme lysis with a phenol/chloroform cleanup. Amplification was performed for four amplicon regions (bacterial 16S rRNA gene regions V3/V4 and V5/V6/V7, Fungal ITS1 and ITS2) in two steps: The first step employed universal amplification primers and oligonucleotide clamps to block host amplification. The second step employed primers consisting of a concatenation of the Illumina adapter P5 (forward) or P7 (reverse), an index sequence (reverse only), a linker region, and the base primer for the region being amplified. Amplicon libraries were quantified fluorescently and products were combined in equimolar concentrations and were sequenced on an Illumina MiSeq lane using a mixture of custom sequencing primers complementary to the linker/primer region of the concatenated primers.

To process the reads, we used the custom pipeline described in Agler et al. (2016). For downstream analyses, operational taxonomic unit (OTU) tables were rarefied to an even depth of reads per sample and summarized to the genus level. A bar chart was plotted including all genera with >5% abundance in any one sample (Supplementary Figures S2 and S3). Beta diversity plots were generated using principal coordinates analyses based on Bray–Curtis similarities between rarefied samples (**Figure 5**; Supplementary Figure S4). Alpha diversity (number of observed taxa) was calculated based on the average of 10 rarefactions of the data (**Figure 5**; Supplementary Figure S4). The raw sequencing data is available on Qiita^2^.

### *Albugo* Strain Determination with Microsatellite Markers

Microsatellite markers were used to analyze genetic diversity between wild *A. laibachii* strains as in Agler et al. (2016). Three primer sets (AlSSR2[F/R], AlSSR6 [F/R], and AlSSR10 [F/R]) were employed which produce amplicons with which *A. laibachii* strains are easily distinguished. PCRs were performed as previously described (Agler et al., 2016) with equal amounts of extracted DNA from endophytic compartment samples as template. Products were visualized on a 3% high-resolution agarose gel (Bio-Budget) with a 100 bp ladder. In some gels weak bands appeared in the background at lengths unexpected for the used markers. These were considered as non-target amplification and only bright bands of similar intensity were analyzed. If the length of amplified bands for all three markers were indistinguishable, we considered strains to be the same.

## Results

### The Secretome of Wild-Grown *A. thaliana* Differs Significantly from Lab-Grown Plants

To get insights into the physiology of wild versus lab-grown plants we used a shotgun proteomics approach to elucidate plant secretomes on plants from two different wild sampling sites and from plants grown in a common garden experiment. We chose two sites nearby Cologne, Germany (Pul and Gey), due to their stable *A. thaliana* population structure and repeated observations of naturally occurring white rust symptoms caused by *Albugo* sp. These sampling sites are located within 2.5 km of one another and at similar elevations such that weather-caused environmental conditions are essentially similar. We sampled leaf material, pooled leaves from several plants at a site, during the early vegetative growth phase of *A. thaliana* in fall before its resting stage over winter and in spring, before it goes into its reproductive stage. All analyses are based on three biological replicates (two spring and one winter) from Pul, and two biological replicates (one spring and one winter) from Gey. We compared wild plants with or without visible white rust infection to lab-grown infected and uninfected mock plants. We focused our analyses on the plant apoplastic space, as it has been shown in previous work that upon biotic and abiotic stress perception there is a massive increase in secretion of defense- or stress-associated proteins into this compartment (Doehlemann and Hemetsberger, 2013; Delaunois et al., 2014). The apoplast is therefore a good analytical readout for plant responses.

**FIGURE 1:**
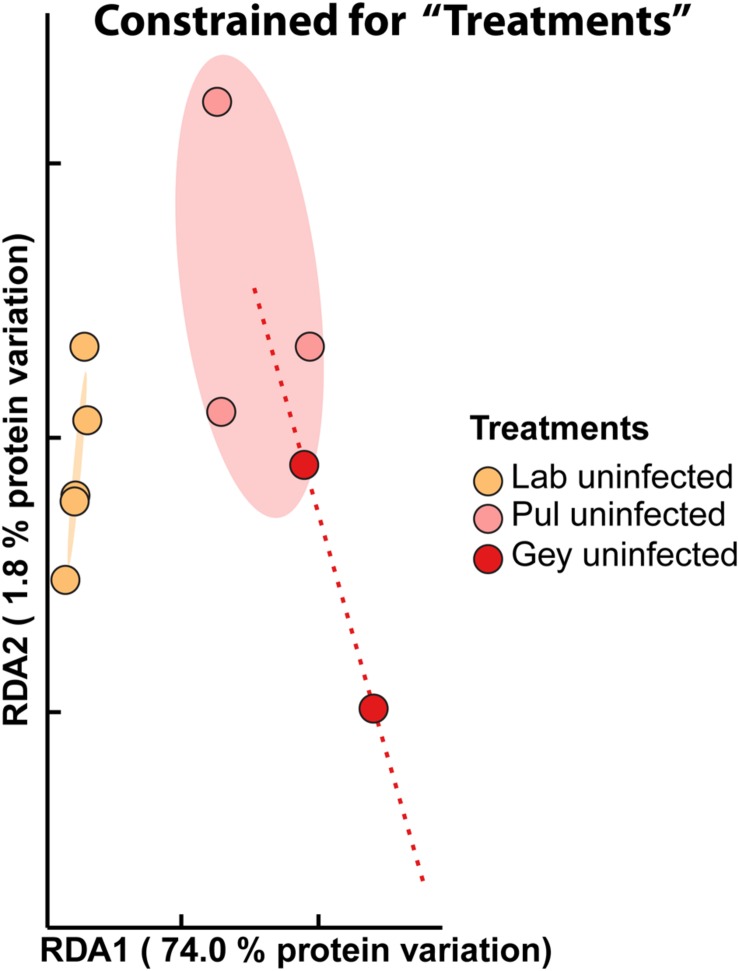
**The secretome of uninfected wild *A. thaliana* differs significantly from *A. thaliana* grown in controlled environment.** The apoplastic fluid proteome (100 most abundant secreted proteins) of asymptomatic wild *A. thaliana* from Pul and Gey clusters significantly different to uninfected lab plants. Seventy-six percent of the variation in protein composition of these samples can be explained by constraining for these different “treatments” [lab uninfected (yellow, *n* = 5), Pul uninfected (pink, *n* = 3), Gey uninfected (red, *n* = 2)] (ANOVA, *p*-value: 0.004). Confidence ellipses (lines) (0.95) based on the standard error show an overlap of Pul and Gey samples indicating their strong similarity.

Analyses revealed between 370 and 585 unique secreted *Arabidopsis* proteins per sample, with abundance values spanning four orders magnitude (Supplementary material: Tables 3–7). We used constrained RDA of the 100 most abundant proteins across all uninfected samples to unravel the factors determining the variation of secretome compositions. Seventy-six percent of the total protein variation (ANOVA based on 999 random class permutations for significance of the treatment constraint, *p*-value: 0.004) was constrained by the factor “treatments,” distinguishing lab, Pul, and Gey samples (**Figure 1**). Here, uninfected lab samples cluster together and are significantly different from wild samples (0.95 confidence interval; **Figure 1**). Samples from different wild sites, however, show significant overlaps of their confidence intervals (**Figure 1**). This demonstrates that wild plants differ significantly from plants grown under controlled conditions even in an uninfected stage and that samples from two different wild sites are more similar to each other than to lab-grown plants.

To check if the phenotypically uninfected wild plants were free of *Albugo* sp. growth, we annotated the protein spectra against the *A. laibachii* Nc14 genome and compared this with laboratory uninfected samples (Supplementary Table S1). Two of the five asymptomatic natural samples (Pul uninfected first and third replicate) exhibited augmented amounts of *Albugo* proteins (11.57% respectively 14.37%), while the other samples were comparable to background levels in non-inoculated control laboratory experiments (maximum 3.07%). The higher levels in the two asymptomatic *Arabidopsis* samples could result from asymptomatic endophytic *Albugo* sp. growth (not visible during sampling) or contamination from *Albugo* spores attached to the leaves. However, they do not cluster closely together with corresponding samples showing white rust symptoms (Supplementary Figure S5C), suggesting that they do not behave like infected samples.

To demonstrate reproducibility of our experimental setup, we performed constrained ordination analyses with the factor “replicates” (three biological replicates) for all lab samples (uninfected and infected). Variation between replicates was not significantly more than random variation (ANOVA, *p*-value: 0.509), indicating the reproducibility of the results under controlled conditions (Supplementary Figure S6). On the other hand, “replicates” was a highly significant factor explaining most of the total variation (58%) in wild samples (for both infected and uninfected samples; ANOVA, *p*-value: 0.004; Supplementary Figure S5C). Not surprisingly, the reproducibility of wild samples is low in comparison to the lab likely because the samples from different sampling dates and seasons (spring/fall) were exposed to different environmental stresses.

**FIGURE 2:**
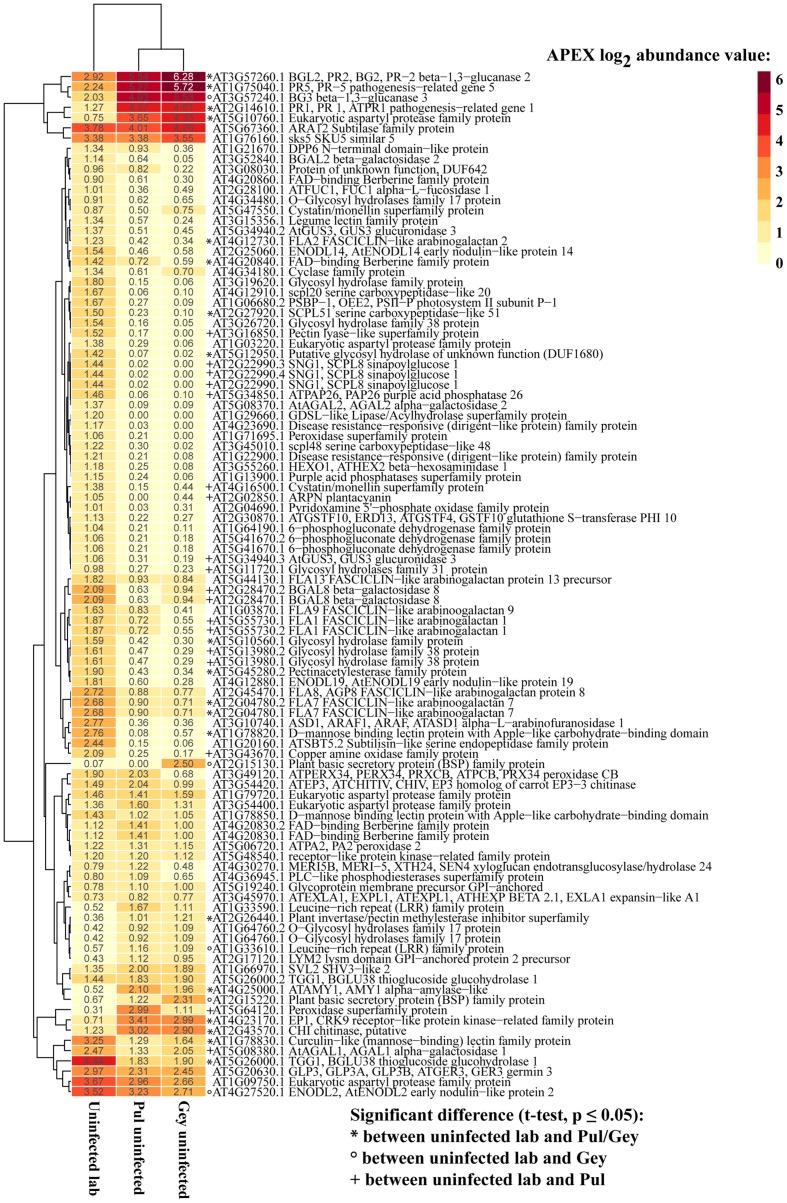
**Phenotypically uninfected wild *A. thaliana* plants have high abundances of defense-related proteins.** Heatmap of the 100 most abundant secreted *A. thaliana* proteins detected in uninfected lab and wild apoplastic fluid samples. The four most abundant proteins were significantly more abundant in wild plants and pathogenesis related. APEX abundance values were averaged across replicates and log_2_ transformed. Significant differences between samples are indicated by symbols.

Considering the overall difference in secretome composition of uninfected wild and lab plants we had a closer look at the 100 most abundant proteins. Forty-two proteins had significantly different abundances between lab plants and wild Pul or Gey plants (Student’s *t*-test, *p*-value ≤ 0.05; **Figure 2**). For uninfected plants, the four overall most abundant proteins in the apoplastic fluid samples (annotated as: PR5 AT1G75040.1, PR1 AT2G14610.1, BG3 AT3G57240.1, PR2 AT3G57260.1) were significantly more abundant in the wild samples and are associated with responses to abiotic or biotic stimulus gene ontology (GO biological process, Berardini et al., 2004; **Figure 2**). In total, twelve proteins that had significantly higher abundance in the wild samples are associated with response to [a]biotic stimulus or to stress (GO biological process). This indicates that phenotypically healthy wild plants have an activated immune system which distinguishes them from plants grown in a controlled lab environment.

### The Oomycete Pathogen *A. laibachii* Does Not Suppress the Activated Immune System of Wild *A. thaliana*

To determine to which extent the oomycete pathogen *Albugo* manipulates its host under controlled conditions and in the wild, we infected *A. thaliana* with two *A. laibachii* strains (isolates Nc14 and MPI1) and an *A. candida* strain (isolate Nc2). The strains Nc14 and Nc2 have been isolated from *A. thaliana* field plantings in Norwich, UK, while the MPI1 strain was the most frequently occurring *Albugo* sp. strain in our common garden experiments in Cologne, Germany (Kemen et al., 2011; McMullan et al., 2015; Agler et al., 2016). Microsatellite markers (van Treuren et al., 1997) demonstrated that in Pul and Gey *A. thaliana* plants were infected with various strains of *A. laibachii* whereas we did not detect *A. candida* with specific primers in any wild or common garden experiment (Supplementary Figure S7 and Agler et al., 2016, for the common garden experiment).

**FIGURE 3:**
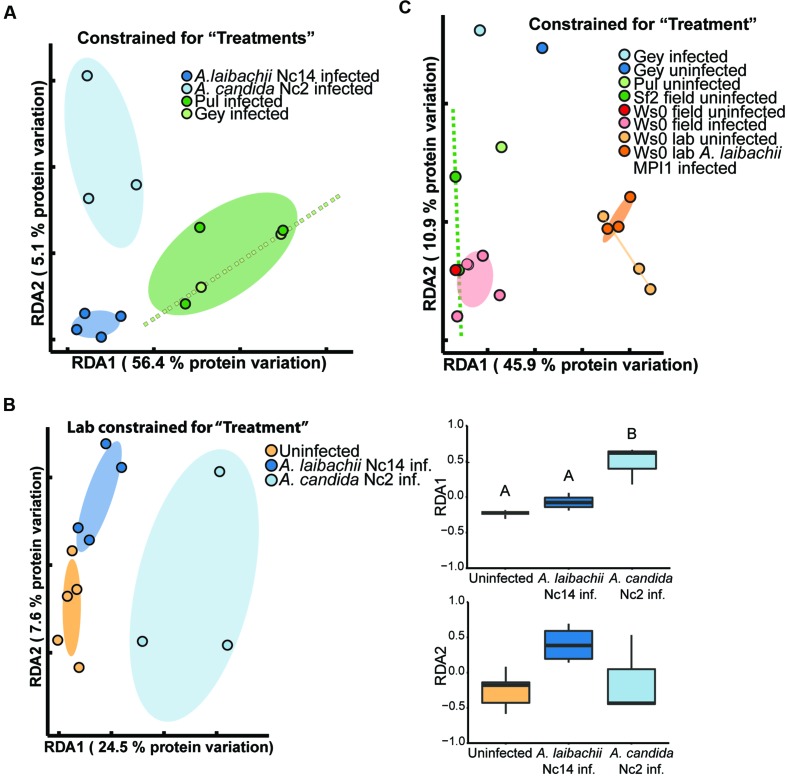
**Wild white rust *Albugo* sp. infections differ from lab infections, but *Albugo laibachii* does not change the *A. thaliana* secretome significantly. (A)** RDA constrained for factor “treatments” with all infected samples. Wild samples from Pul (dark green, *n* = 3) and Gey (light green, *n* = 2) cluster significantly different to laboratory infections of *A. laibachii* Nc14 (dark blue, *n* = 4) and *A. candida* Nc2 (light blue, *n* = 3). Confidence ellipses are based on standard error. **(B)** RDA constrained for factor “treatments” with all laboratory samples. Uninfected (yellow, *n* = 5) and *A. laibachii* Nc14 (dark blue, *n* = 4) infected samples cluster closely together apart from *A. candida* Nc2 (light blue, *n* = 3) infected samples. The spread of the protein samples on both axes plotted in boxplots indicates a significant difference between *A. candida* Nc2 and *A. laibachii* Nc14/uninfected samples along RDA1-axis with 24.5% of the total variation (Tukey honestly significant difference (HSD), *p*-value < 0.05). (C) *Arabidopsis thaliana* Ws-0 grown in a common garden experiment under wild conditions (red, asymptomatic plants; pink, *Albugo*-infected plants) has significantly different secretome compositions compared to *A. thaliana* Ws-0 grown under laboratory conditions (yellow, uninfected; orange, *Albugo*-infected). Confidence ellipses (0.95) are based on standard error. All samples presented in **(C)** were analyzed with a Q Exactive Plus following in-solution digestion (see liquid chromatography tandem mass spectrometry (LC-MS/MS) Analysis).

Constrained analyses revealed clear differences in protein composition of the infected samples: 63% of the total protein variation was constrained by distinguishing the samples from Pul, Gey, and the two *Albugo* lab infections (factor “treatment”; ANOVA, *p*-value: 0.015; **Figure 3A**). Pul and Gey infections clustered closely together with significant separation from both *A. laibachii* Nc14 and *A. candida* Nc2 infected samples in the laboratory (**Figure 3A**). Even though the wild samples were from spatially separated sampling sites and infected with different *A. laibachii* strains, there were no significant differences in their secretome composition. Comparable to the apoplastic secretome of uninfected and asymptomatic *A. thaliana* plants (see The Secretome of Wild-Grown *A. thaliana* Differs Significantly From Lab-Grown Plants), wild plants showing a white rust phenotype differ significantly from plants showing white rust symptoms following infections under controlled lab conditions.

To unravel to which extent *Albugo* sp. can change the *A. thaliana* protein secretion following successful colonization, we compared for each environmental condition (laboratory/wild) the secretome of plants showing successful infection of *Albugo* with uninfected plants. Under laboratory conditions, 32% of the total variation were constrained by separating uninfected, *A. candida* Nc2 infected and *A. laibachii* Nc14 infected samples (ANOVA, *p*-value: 0.013; **Figure 3B**). All uninfected/symptomless samples clustered closely together with *A. laibachii* Nc14 infected leaf samples, apart and with no significant overlap with *A. candida* Nc2 infected samples. Considering the spread of the plotted samples along RDA1 (*x*-axis; explains 24% of the protein variation) there is a significant difference between the *A. candida* Nc2 and uninfected/*A. laibachii* Nc14 infected samples (Tukey HSD, *p*-value: 0.001; **Figure 3B**). The uninfected and *A. laibachii* Nc14 infected samples cluster closely together without significant differences along this axis and with only weak differences along the *y*-axis. Therefore, under controlled conditions *A. laibachii* Nc14 infections had non-significant effects on the abundance of secreted *A. thaliana* proteins.

In order to better unravel the environmental influences on *Arabidopsis* secretomes and to check whether differences between lab and field were due to differences between Ws-0 used in the lab and wild plants, we planted *A. thaliana* Ws-0 (*Albugo* susceptible) and Sf-2 (*Albugo* resistant) plants, in a common garden experiment (described in Agler et al., 2016). Nearly all wild-grown *A. thaliana* Ws-0 plants were infected in spring (mostly by *A. laibachii* strain MPI1) and we compared these to laboratory experiments with an isolate of this *Albugo* strain. The high infection rates of Ws-0 impeded sampling of asymptomatic plants. RDA constrained for “treatments” (ANOVA, *p*-value: 0.001) showed a significant difference between the secretomes of field- and lab-grown *A. thaliana* Ws-0. Under laboratory conditions, *A. thaliana* Ws-0 plants infected with *A. laibachii* MPI1 did not significantly differ from uninfected plants. This was comparable to field-grown *A. thaliana*, which showed dense clustering of samples irrespective of infection status (**Figure 3C**). *Albugo*-resistant *A. thaliana* Sf-2 samples clustered closely with *A. thaliana* Ws-0. The single replicate from Pul and Gey that was analyzed with the same method clustered away from the plants of the common garden experiment but were more variable. Therefore, these results confirmed differences that were observed between lab-grown plants ((un-)infected) and wild *Arabidopsis* samples from Pul and Gey (**Figures 1** and **3A**).

**FIGURE 4:**
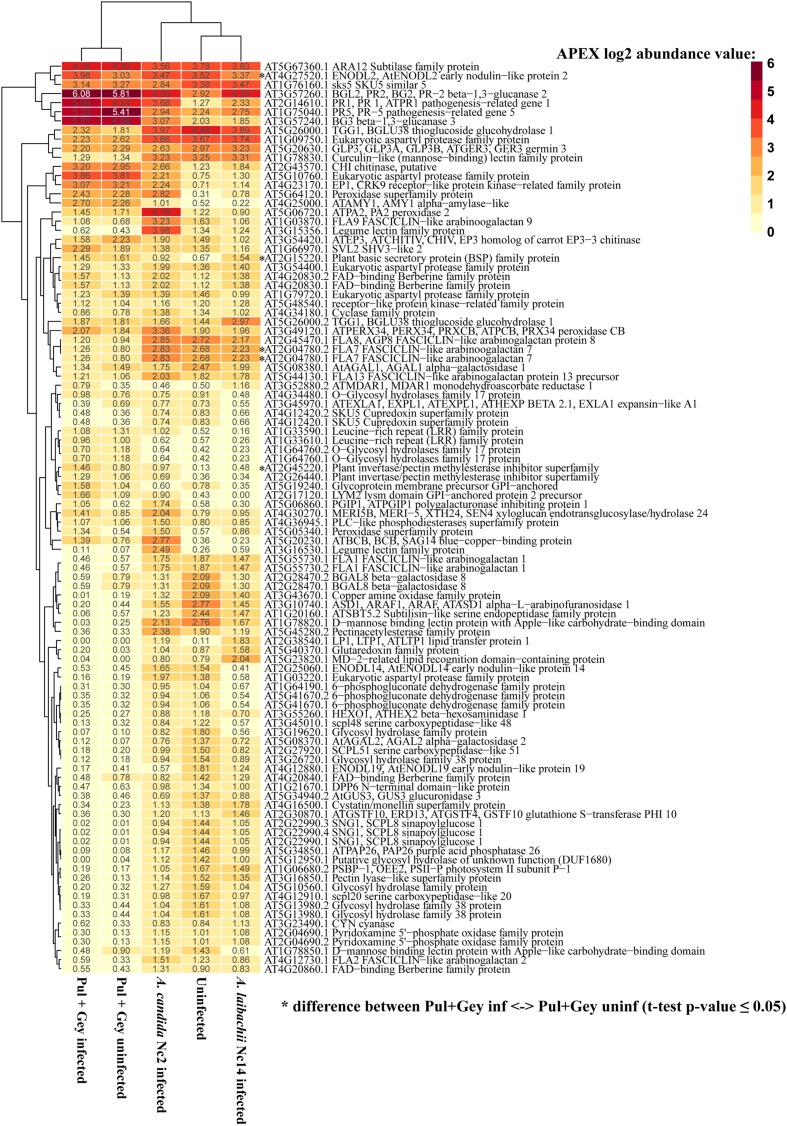
**Asymptomatic, uninfected wild *A. thaliana* plants show an activated immune system, which is not changed by *Albugo* sp. infections.** The most abundant *A. thaliana* proteins in wild *Albugo* sp.-infected and -uninfected plants are related to defense responses. APEX abundance values were averaged across replicates and log_2_ transformed. Significant differences between wild samples are indicated by symbols.

Constraining for differences between infected and uninfected samples by site (i.e., within each of Pul and Gey) explained 24% of the variation in secretome composition, but this was not significant (ANOVA, *p*-value: 0.744; Supplementary Figure S5A). Similarly, constraining for infection status across both natural sites, which only distinguishes all plants with white rust symptoms from symptomless plants, explained 6% of the total protein variation and was not significant (ANOVA, *p*-value: 0.686; Supplementary Figure S5B). This indicates that similar to results in the lab, the protein composition in *A. thaliana* apoplastic fluid is highly similar in *A. laibachii*-infected and -uninfected wild plants (overlapping confidence ellipses based on standard error; Supplementary Figure S5A). Similar to uninfected wild plants (**Figure 2**), *A. laibachii*-infected wild plants exhibited high levels of PR proteins like beta-1, 3-glucanases (PR2 AT3G57260.1, AT3G57240.1), PR1 (AT2G14610.1) and PR5 (AT1G75040.1; APEX log_2_ abundance > 5.0; **Figure 4**). Since there was no significant difference (paired *t*-test, *p*-value ≥ 0.05) in abundance of these proteins in uninfected wild plants compared to wild plants showing white rust symptoms, *A. laibachii* infection does not significantly affect *A. thaliana* defense protein secretion. Furthermore, *A. laibachii* infections do not reduce the secretion of defense proteins that were already present in plants prior to infection. These results suggest that the obligate biotroph *A. laibachii* is adapted to *A. thaliana* triggered immune responses in the wild and can complete its infection cycle without severe suppression of the apoplastic protein based defense machinery.

### Bacterial Alpha Diversity Changes with *Albugo* sp. Infection While Fungal Community Compositions Are More Stable

To unravel which biotic factors might have triggered the observed elevated levels of PR proteins under field conditions regardless of *Albugo* infection status, we characterized the endophytic microbial communities of *Albugo* sp. infected versus uninfected *A. thaliana* plants in Pul and Gey via amplicon sequencing (bacterial 16S rRNA, fungal ITS).

**FIGURE 5:**
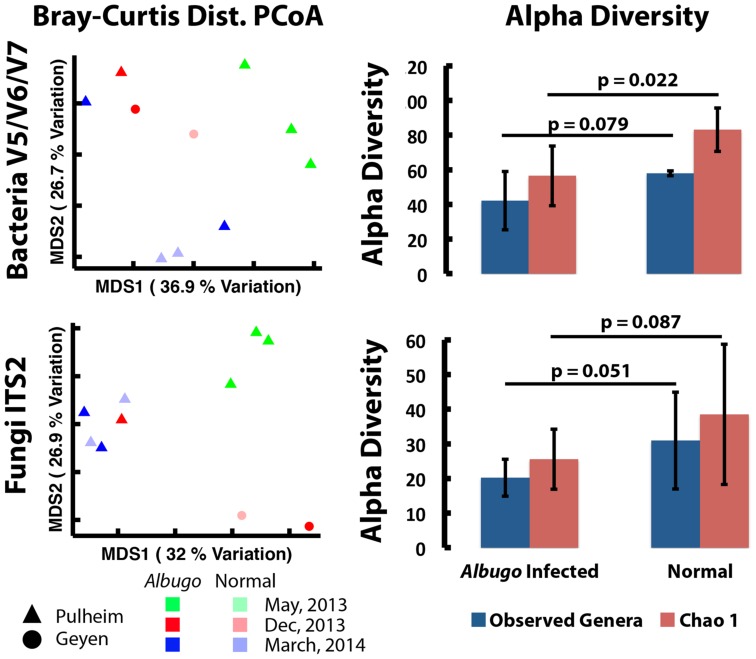
**Unconstrained ordination of bacterial and fungal communities and their respective alpha diversity.** Principle component analyses of microbial communities from Pul and Gey, based on amplicon sequencing (bacterial 16S rRNA V5/V6/V7 region, fungal ITS2 region). The alpha diversity within bacterial and fungal communities decreases in infected plants.

The sampling was done at the same time points as for proteomics and the processing was described in Agler et al. (2016) (see Amplicon Sequencing of Microbial Communities). Unconstrained ordination of the infected and uninfected wild samples indicated that the bacterial communities were fairly variable and clustered by the sampling time point (**Figure 5**; Supplementary Figure S4). Similar to fungi, bacterial communities grouped by plant generations (the samples that were harvested in December 2013 and March 2014 are one plant generation since *A. thaliana* germinates in fall, is vegetative over winter and dies in late spring following reproduction; **Figure 5**; Supplementary Figure S4). Contrary to the bacterial communities, only the fungal community in Gey clusters separately from Pul samples, supporting previous results suggesting that fungi are more location-specific than bacteria (Agler et al., 2016; Coleman-Derr et al., 2016). Endophytic fungi, detected at Pul or Gey, belonged largely to the order Pleosporales or the class of Leotiomycetes, which are, often necrotrophic, ascomycotal fungi (Supplementary Figure S3). Calculating the alpha diversity within the bacterial and fungal communities in the infected and uninfected samples revealed a decrease of the diversity in infected plants (**Figure 5**; Supplementary Figure S4).

Taken together the results indicate that *Albugo* sp. infections have a stronger influence on the colonization of bacteria where sample clustering is more variable and *Albugo*-infection status correlates to diversity (Agler et al., 2016). We therefore propose that fungi trigger the observed host immune responses, since these communities are similar in infected and uninfected samples and therefore seem to be less under the control of *Albugo*.

### *Albugo laibachii* Infections Are Less Affected by Altered Host Hormone Levels than *Hpa*

*Arabidopsis thaliana* immune responses to biotic stresses often result in alteration of plant hormone levels including SA-mediated defense against (hemi-)biotrophs or jasmonic acid (JA)/ethylene-mediated defense against necrotrophs (Thomma et al., 1998; Glazebrook, 2005). Especially PR1, PR2, and PR5 proteins were shown to be SA responsive (Uknes et al., 1992), and we showed that they were highly abundant in all wild *A. thaliana* samples. *Hpa* has an overlapping host range and is a natural competitor of *Albugo* sp. on *A. thaliana*, because as obligate biotrophic oomycetes they occupy a similar niche.

**FIGURE 6:**
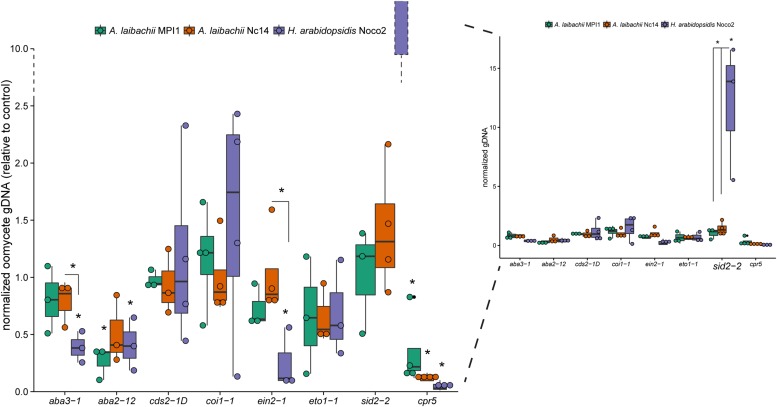
**Oomycete growth quantification via qPCR in different *A. thaliana* hormone mutants.** Oomycete growth (*A. laibachii* MPI1/Nc14, *H. arabidopsidis* Noco2) was quantified via qPCR at 10 dpi (*A. laibachii*) or 5 dpi (*H. arabidopsidis*) in different *A. thaliana* hormone mutants (Col-0 as control). *A. laibachii* Nc14 growth (orange) was only significantly different to the control in *cpr5* mutants. *H. arabidopsidis* Noco2 growth (purple) was affected in *aba3-1*, *aba2-12*, *ein2-1*, *sid2-2*, and *cpr5* mutants. Asterisks indicate significant differences to Col-0 control infections and differences of infections within a mutant group (*t*-test, *p* < 0.05, *n* = 3 or 4).

During our experimentation, we phenotypically observed far more *A. laibachii* infection in the wild than other biotrophic pathogens of *A. thaliana* like *Hpa*. Therefore, we wanted to know if *A. laibachii* is better adapted to the primed immune system (i.e., constantly high PR protein levels) than *Hpa*. We compared both pathogens in growth assays on *A. thaliana* hormone mutants for their infection efficiency (**Figure 6**). Oomycete growth quantification via qPCR showed that *Hpa* Noco2 is especially affected in ABA biosynthesis-deficient mutants (*aba3-1*, *aba2-12*, negative effect), an SA-induction deficient mutant (*sid2-2*, positive effect) and a mutant with constitutive expression of PR-genes (*cpr5*, positive effect) compared to *A. laibachii.* Furthermore, *Hpa* Noco2 growth is significantly lowered in the *ein2-1* mutant (ethylene insensitive), which has elevated JA levels after pathogen treatment and constitutive *PR1* expression (Penninckx et al., 1998; Chen et al., 2009). Two different *A. laibachii* strains (MPI1 and Nc14) were tested. Contrary to *Hpa*, *A. laibachii* Nc14 and MPI1 colonization was very resilient, since its growth was only affected (relative to mock control) in the *cpr5* (Nc14/MPI1) and the *aba2-12* (MPI1) mutant backgrounds. In total, *Hpa* Noco2 growth varied more in the different mutant backgrounds (significant difference to mock control in five of eight mutants), than *A. laibachii* (significant difference in one of eight mutants for Nc14 and two of eight mutants for MPI1). This suggests that *A. laibachii* strains show a high plasticity to adapt to a broad range of host pre-existing defense fluctuations. This plasticity might give an advantage in competing for limited growth space in nature.

## Discussion

*Arabidopsis thaliana* is the best-studied flowering plant under controlled lab conditions (Koornneef and Meinke, 2010). How *A. thaliana* physiology changes and what happens on a molecular level under natural conditions compared to lab conditions is largely unknown. We have addressed this knowledge gap by comparing the apoplastic secretome of *A. thaliana* plants grown under standard experimental conditions in the lab with wild plants in stable, well-established, populations or with wild-grown plants in a common garden experiment. Our data demonstrates that morphologically healthy, wild- or field-grown *A. thaliana* plants are significantly different in physiology from plants grown under controlled lab conditions. The main difference is that naturally grown plants have significantly higher abundances of defense- and stress-related proteins in the apoplastic space (**Figure 2**).

### Asymptomatic Wild *A. thaliana* Has an Induced Immune System

The apoplastic space is important for plant defense and pathogen virulence, as it is one of the main contact points of the host to invading pathogens (e.g., Kaffarnik et al., 2009; Floerl et al., 2012; Ali et al., 2014; Kim et al., 2014) and beneficial endophytes (e.g., Dong et al., 1994; Chi et al., 2005). Colonizing pathogens are generally recognized by plant cells in the apoplast where they are directly attacked by plant defenses. Upon microbe recognition, plants try to limit their growth by, e.g., reacting with a burst of reactive oxygen species (ROS; Daudi et al., 2012) or producing antimicrobial proteins from the PR-family (PR proteins; Loon et al., 2006). In apoplastic fluids extracted from wild, asymptomatic *A. thaliana* plants we found the most abundant proteins were PR2 (pathogenesis-related 2), PR5, BG3 (beta-1,3-glucanase 3), PR1 and eukaryotic aspartyl protease, which indicates triggered immune responses. Especially PR1, PR2, and PR5 are known marker proteins for SA-dependent systemic acquired resistance (SAR; Uknes et al., 1992, 1993), which is a long-lasting form of broad-spectrum disease resistance against avirulent pathogens in the whole plant (Maleck et al., 2000). High abundance of PR1 and SAR often goes along with redox regulation and accumulation of ROS, which are generated by nicotinamide adenine dinucleotide phosphate (NADPH) oxidases or apoplastic peroxidases (Mammarella et al., 2015). *A. thaliana* encodes several peroxidases, of which the apoplastic peroxidase 34 (PRX34, AT3G49120.1) is specifically activated under plant defense conditions, as well as peroxidase 33 (PRX33, AT3G49110.1; Daudi et al., 2012; Mammarella et al., 2015). However, PRX34 and PRX33 peroxidases were not significantly different in abundance in lab or wild plants and PRX33 was generally only very low abundant (not among top 100 proteins, **Figure 4**). A further 16 extracellular class III peroxidases that were shown to be expressed in leaves (Welinder et al., 2002), were either not detected or only at very low abundances with no difference between symptomless wild and lab samples. This could suggest that no ROS-burst or hypersensitive response was triggered in asymptomatic wild plants. In lab-grown unchallenged plants, only low levels of PR-proteins were detectable, consistent with the basal level that has been described for *A. thaliana* in previous studies (Floerl et al., 2012; Trentin et al., 2015).

The most abundant protein in untreated lab grown samples is a thioglucoside glucohydrolase (known as myrosinase; AT5G26000.1), which is significantly more abundant compared to asymptomatic field samples (**Figure 2**). Glucosinolates are secondary metabolites that can be cleaved by the enzyme thioglucoside glucohydrolase resulting in toxic products against fungi and insects (Barth and Jander, 2006; Halkier and Gershenzon, 2006; Bednarek et al., 2009). As such it can deter generalist herbivores, but might attract crucifer specialists (Barth and Jander, 2006). Taking lab-uninfected *A. thaliana* protein abundances as a measure for protein levels under unstressed conditions, we hypothesize that microbes colonizing healthy, uninfected wild plants lead to suppression of myrosinases and glucosinolates in nature. Thus, defense against eukaryotic microbes might be lowered, facilitating the colonization of fungi and oomycetes in nature and ultimately leading to the induced immune system.

### Restructuring the Leaf Microbial Community by the Microbial Hub *A. laibachii* Is Not Mediated via Host Protein Secretion

Naturally grown *A. thaliana* plants accommodate a broad range of microbes (Vorholt, 2012; Agler et al., 2016), which are probably responsible for the observed activated defense and myrosinase suppression. Thus, our goal was to dissect the endophytic microbial community to identify responsible key organisms. Our community profiling revealed that bacteria did not differ markedly between the two wild populations of Pul and Gey, whereas fungal communities showed a significant location specificity. This indicates a rather homogenous spread of bacterial inoculum, while fungal dispersal/growth is more unequal and site specific. This agrees with previous reports that bacterial communities vary in their relative abundance of species between sites, while fungal communities differ by presence/absence between different sites (Agler et al., 2016; Coleman-Derr et al., 2016). Bacterial and fungal communities were fairly stable over one plant generation (December 2013, March 2014) even though challenged by natural biotic and abiotic fluctuations, but differed significantly from one to the next plant generation (May 2013).

Endophytic fungi belonged largely to the order Pleosporales, which has many members that are necrotrophic plant pathogens like the detected *Alternaria* sp., *Ascochyta* sp. or *Boeremia* sp. Even though these fungi were detected as endophytes via sequencing, the plants did not show any signs of necrosis and were otherwise healthy during sampling. Possibly, these fungi initially colonized plants and triggered the observed immune responses and the primed host immune system restricted their growth. Although *Alternaria brassicicola* was shown to trigger SA-marker gene expression, growth restriction, and resistance against *A. brassicicola* relies on callose-deposition (Ton and Mauch-Mani, 2004). Even though we did not observe the callose synthase enzymes (e.g., AtGSL5), we cannot exclude their activation, since they are cellular and not detectable in the apoplast.

Besides fungi, the most abundant eukaryotic microbes observed in wild *A. thaliana* populations were the causal agents of white rust symptoms, *Albugo* sp. Endophytic bacterial communities of wild *A. thaliana* plants changed after infection by *Albugo* sp. showing significantly reduced alpha diversity and Gammaproteobacteria (mostly *Pseudomonas* sp.) dominating bacterial communities. Again, this is consistent with Agler et al. (2016), which highlighted an *A. laibachii*-mediated reduction of the bacterial alpha diversity of endo- and epiphytes and a stabilization of the community structure. Identifying *A. laibachii* as a hub organism structuring the *A. thaliana* microbial community left one main question: Does the hub reduce bacterial diversity via triggering plant defense or is there a direct interaction between hub and microbes? With this work, we can now show that the increase of relative abundance of major bacterial taxa and limiting the bacterial diversity is not mediated via the host protein secretion, as the hub microbe *A. laibachii* does not influence the *A. thaliana* protein secretion significantly. This suggests that direct microbe–microbe interactions might take place in the apoplastic space that result in observed decreased diversity of bacteria. Investigating the secretome of *Albugo* sp. during apoplastic space colonization could better resolve such microbe–microbe interactions.

### *Albugo laibachii* Tolerates Apoplastic Broad-Spectrum Immune Responses Instead of Suppressing Them

Even though *A. laibachii* was shown to suppress resistance-gene-mediated broad-spectrum resistance (non-systemic; Cooper et al., 2008), we showed that this does not translate into broad effects on suppressing apoplastic defense. Thus, *Albugo* sp. are still able to go through their whole infection cycle without suppressing already activated host immune responses in the apoplast. In controlled lab conditions, where replicability was significantly higher, revealed that *A. laibachii* Nc14-infections lowered the protein abundance of only four *A. thaliana* proteins (serine carboxypeptidase-like protein, pectinacetylesterase family protein, mannose binding lectin protein and alpha-galactosidase; Supplementary Figure S8). Two of these proteins, pectinacetylesterase and alpha-galactosidase, could be involved in cell wall organization processes and could therefore be *A. laibachii* target proteins for haustoria formation in the host cells. The other two proteins, serine carboxypeptidase and mannose binding lectin protein, might contribute to plant defense reactions as carboxypeptidases are involved in protein degradation and many lectins bind foreign polysaccharides and can play a role in pathogen recognition (Lehfeldt et al., 2000; Sharon and Lis, 2004). Nevertheless, *A. laibachii* has suppressive effects on the abundance of only 4% of the host secreted proteins, none of which are classics of defense, like PR proteins or peroxidases (Supplementary Figure S8). Therefore, one might speculate that instead of broad-spectrum suppression, *Albugo* must have developed some mechanisms to protect its hyphae from immune responses. Similar mechanisms have been observed for fungal pathogens that use α-1-3-glucan to protect chitin in the cell wall from degradation by plant chitinases (Oliveira-Garcia and Valent, 2015).

Being adapted to the naturally occurring immune responses in wild host plants and not triggering further defense reactions seems to be advantageous in competing for limited growth space. *A. laibachii* appears to not only tolerate host-mediated broad-spectrum immune responses but also its growth is very resilient in host plants with altered hormone levels. On the other hand, the potential competitor *Hpa* – a common obligate biotroph pathogen on *A. thaliana* in the wild (Holub et al., 1994) is strongly affected (positively and negatively) by hormonal changes. The *cpr5* mutant secretome might be most similar to wild *A. thaliana* plants as it has high protein concentrations of PR1, PR2, PR5 and the defensin protein PDF1.2 (Bowling et al., 1997). The *cpr5* mutant also harbors high levels of SA compared to Col-0 wild type plants and was shown to be fully resistant against *Hpa* Noco2 (Bowling et al., 1997; Clarke et al., 2000). Infection screens were formerly based on conidiophores or spore counts at 7 dpi (Bowling et al., 1997; Clarke et al., 2000). We also did not observe sporulation after *Hpa* Noco2 infections at 5 dpi, but the more sensitive qPCR quantification revealed low levels of *Hpa* growth (**Figure 6**). *A. laibachii* infections were routinely harvested at 10 dpi and showed weak white rust sporulation in *cpr5* mutants, as well as stronger quantified growth than *Hpa*, highlighting the capability of *A. laibachii* to grow even under induced plant defense. Furthermore, *Hpa* infections were affected in *aba2-12*, *aba3-1*, *ein2-1*, and *sid2-2* mutant background while *A. laibachii* was not. The *sid2-2* mutant, defective in ISOCHORISMATE SYNTHASE 1, does not accumulate pathogen-inducible SA and is impaired in its SAR activation (Nawrath and Métraux, 1999; Bernsdorff et al., 2015). Thus the *sid2-2* mutant is hyper susceptible to *Hpa* infections, whereas *A. laibachii* grew only slightly better on these plants (**Figure 6**; Nawrath and Métraux, 1999). The mutants that are impaired in ABA biosynthesis (*aba3-1*, *aba2-12*) did not affect *A. laibachii* Nc14 growth (*A. laibachii* MPI1 was affected in *aba2-12*), but *Hpa* Noco2 grew significantly less in all of these (**Figure 6**; Léon-Kloosterziel et al., 1996; Adie et al., 2007). The impairment of ABA synthesis results in decreased levels of JA and increased levels of SA-induced genes after pathogen infection, compared to wild type (Adie et al., 2007). Together, our results imply different strategies of the two pathogens: *Hpa* can quickly take advantage of suppressed defense where it probably would outcompete *A. laibachii*, but it is also more susceptible to plant defenses than the highly plastic and robust *Albugo*. Probably the latter situation is more common in many plant populations, explaining our observation of less *Hpa* in the wild.

**FIGURE 7:**
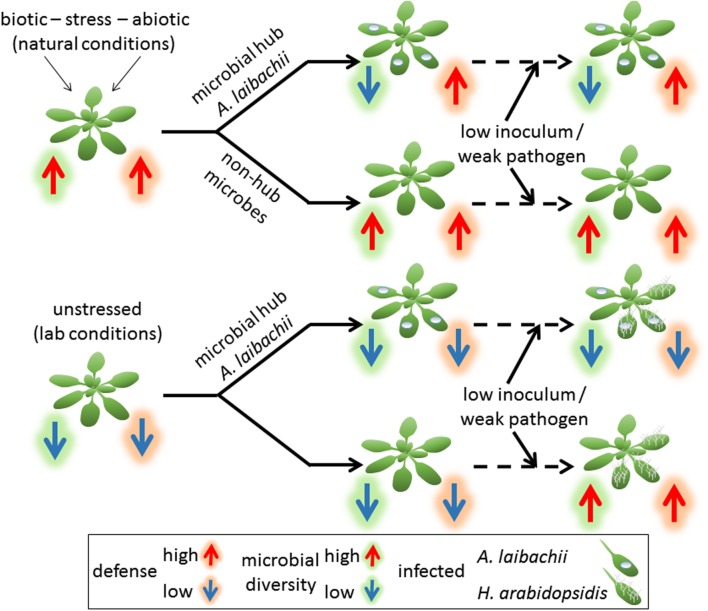
**The well-adapted microbial hub *Albugo* keeps host immunity active to gain colonization advantages over competitors in nature.** Under natural growth conditions, *A. thaliana* shows an induced immune system and a high microbial diversity in the phyllosphere. Infections of *A. laibachii* influence the microbial community structure, but do not suppress host defense reactions so that colonization of weak pathogens is hindered. If the *A. thaliana* immune system is not induced, as in plants grown under lab conditions, pathogens like weak strains of *H. arabidopsidis* can infect *A. thaliana* even after a preceding *Albugo* infection. *Albugo* does not trigger strong host defense reactions, which allows the growth of *H. arabidopsidis.*

In summary, our work gives significant new experimental insight into how plants behave under natural conditions on a molecular level and dissects clear, significant differences to experiments under controlled lab conditions (**Figure 7**). It further indicates that oomycete pathogens of the genus *Albugo*, which are important microbial hubs regulating the *A. thaliana* microbial community in the wild, are extremely fine-tuned to neither trigger strong host defense reactions, nor to act on broad-spectrum defense suppression in the apoplast. Therefore, we hypothesize that *Albugo* is well adapted to an active host immune system, which gives them an advantage over competitors in fighting for limited growth space in the same niche.

## Author Contributions

Experiments were conceived and designed by JR, MA, IF, and EK. The experiments were performed by JR, MA, AP, KK, and IF. Data analysis was conducted by JR and MA. The manuscript was written by JR and EK. All authors corrected the manuscript and discussed the data.

## Conflict of Interest Statement

The authors declare that the research was conducted in the absence of any commercial or financial relationships that could be construed as a potential conflict of interest.

## Footnotes

1 http://www.maxquant.org

2 https://qiita.ucsd.edu/; study 10369
