# Supplementary material for: Obligate Biotroph Pathogens of the Genus Albugo Are Better Adapted to Active Host Defense Compared to Niche Competitors
Source: Front Plant Sci. 2016 Jun 20;7:820. doi: 10.3389/fpls.2016.00820 (PMC4913113; doi:10.3389/fpls.2016.00820)
Supplement: Supplementary file 7 [file Data_Sheet_1.PDF]

Supplementary Figure S1 The *A. thaliana* Ws-0 secretome differs significantly under different growth conditions. (A) Samples marked with an asterisks were excluded for the analysis in figure 3C based on their low correlation to the respective replicates (B). The excluded samples had significantly lower MS/MS identification rates (Pul_inf, Sf2_noninf_2, Ws0_field_uninf_2.1, Ws0_field_uninf_2.2) compared to all other samples or had a significantly higher MS/MS identification rate (Ws0_lab_MPI1_inf_4) (data not shown). (B) Pearson correlation matrix of LFQ intensities shows clustering into four groups: Two clusters of samples with low correlation coefficients that were excluded for further analyses and two clusters distinguishing field-grown plants from lab-grown plants. (A+B) All samples were analyzed with a Q Exactive Plus following in-solution digestion (Method 2.5).

Supplementary Table S1 Overview of detected *Albugo* proteins in *A. laibachii* infected and uninfected samples. The ratio of detected *A. laibachii* proteins per *A. thaliana* proteins was calculated with all proteins detected in apoplastic fluid samples. Pul uninf 1 and 3 show higher ratios of *Albugo* proteins compared to laboratory samples, which indicates asymptomatic *Albugo* growth in these samples. All samples were analysed with a LTQ Velos following in-gel digestion (Method 2.5, 2.6).

Supplementary Table S2 Overview of detected *Albugo* proteins in *A. laibachii* infected and uninfected samples. The ratio of detected *A. laibachii* proteins per *A. thaliana* proteins was calculated with all proteins detected in apoplastic fluid samples. All asymptomatic wild-grown plants have, comparable to lab-grown plants, low ratios of *Albugo* proteins. All samples were analyzed with a Q Exactive Plus following in-solution digestion (Method 2.5, 2.6).

Supplementary Figure S2 Bacterial genera that make up at least 10% of reads in any one sample. Data is based on relative abundance calculated from data that was not first subsampled.

Supplementary Figure S3 Fungal genera that make up at least 10% of reads in any one sample. Data is based on relative abundance calculated from data that was not first subsampled.

Supplementary Figure S4 Unconstrained ordination of bacterial and fungal communities and their respective alpha diversity. Principle component analyses of microbial communities from Pul and Gey, based on amplicon sequencing (bacterial 16S rRNA V3/V4 region, fungal ITS1 region), show the separation of bacterial communities by plant generations. The alpha diversity within bacterial communities decreases in infected plants. Fungal communities separate by location (Pul/Gey). The alpha diversity in fungal communities decreases significantly with *Albugo* infection.

Supplementary Figure S5 Asymptomatic wild plants do not differ significantly from plants with white rust symptoms. (A) The protein secretion in natural infected (dark/light green) or uninfected (dark/light red) plants (Pul and Gey) does not differ from one another. Constraining for treatment is not significant (ANOVA, p-value: 0.744), indicated by overlapping confidence intervals. (B) Separating the wild samples via constrained analysis by "infection status" with distinguishing asymptomatic plants (purple, n=5) from plants with white rust symptoms (orange, n=5) explained only 6.4 % of the protein variation (nonsignificant, ANOVA, p-value: 0.686). One Gey infected samples clusters within the confidence interval of uninfected samples, indicating their strong similarity. (C) Wild samples from different time points (one= March 2013, two= December 2013, three= May 2014)

cluster largely apart from each other in constrained analyses (ANOVA, p-value: 0.004) and explain 58 % of the total protein variation. Different replicates vary by color and treatments by shape. (A+B+C) All Pul and Gey samples were analyzed with a LTQ Velos following in-gel digestion (Method 2.5, 2.6).

Supplementary Figure S6 Analyses of the apoplastic secretome under laboratory conditions is highly replicable. Constrained analysis with the factor “Replicates”, of the apoplastic fluid proteome (100 most abundant secreted proteins) of *Albugo* sp. infected and uninfected plants conducted in the lab, was not significant (ANOVA, p-value: 0.509). Confidence ellipses (lines) (0.95) based on the standard error show an overlap of samples from different biological replicates, which indicates their strong similarity. Different replicates are indicated by colors, while treatments differ by shape.

Supplementary Figure S7 Relative diversity of observed *Albugo laibachii* strains causing white rust symptoms on *A. thaliana* in Pul and Gey. *A. laibachii*- specific microsatellite markers were used for the analysis of *Albugo* strain diversity at the wild sites Pulheim (Pul) and Geyen (Gey). Both sites show no overlap in detected *Albugo* strains and Gey showed a bigger diversity of detected strains. Different detected *Albugo* strains are indicated by colors.

Supplementary Figure S8 *Albugo* sp. influence the abundance of a minority of *A. thaliana* secreted proteins. Heatmap with the 100 most abundant secreted *A. thaliana* proteins in uninfected and *Albugo* sp. infected apoplastic fluid samples under lab conditions. APEX abundance values are averaged across replicates and $\log_2$ transformed. Significant differences between *Albugo* sp. infected and uninfected samples are indicated by symbols.

Supplementary Figure S1

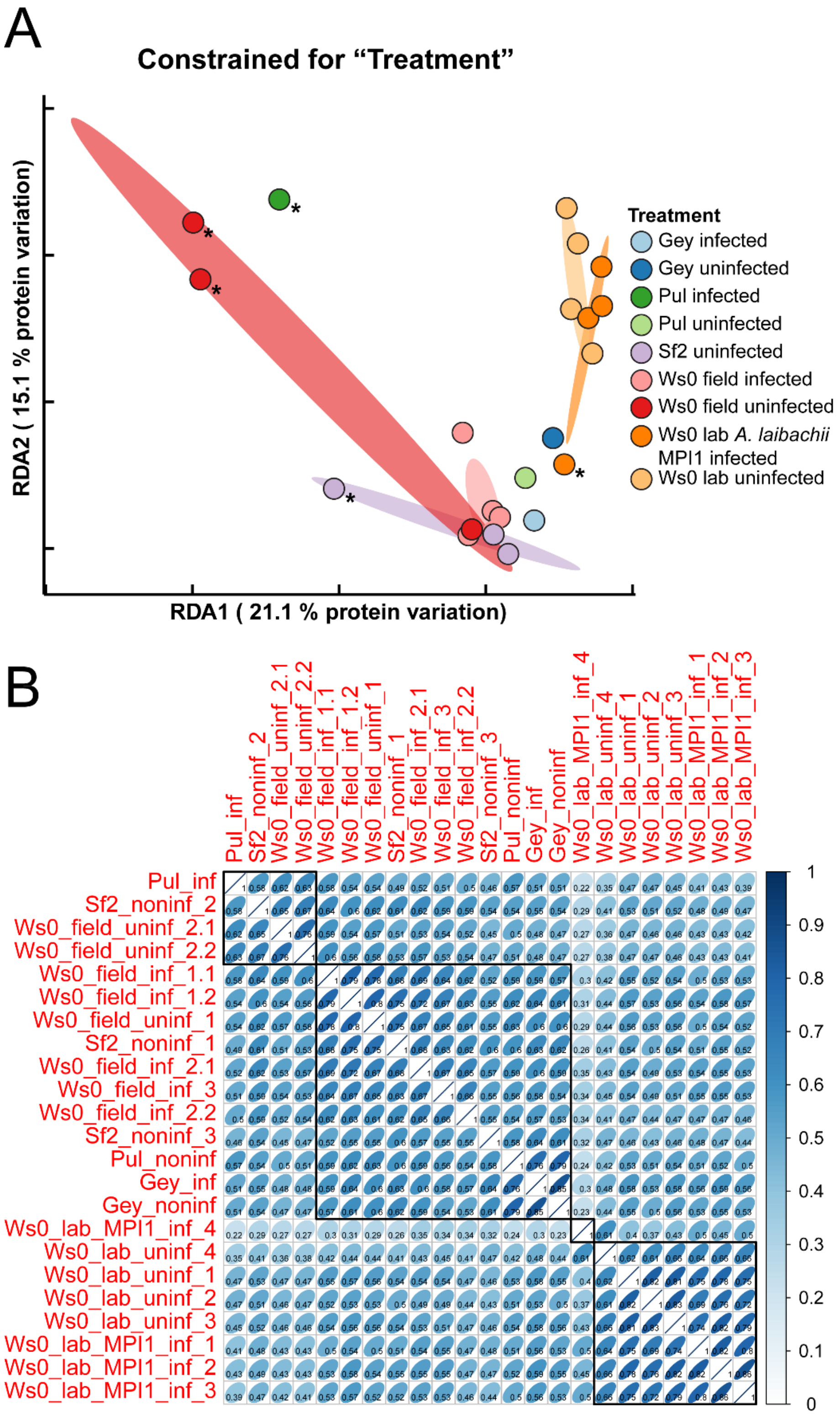

Supplementary Table S1

Sample	Number of *Albugo* proteins	Number of *A. thaliana* proteins	*Albugo* / total proteins	**Ratio uninf/inf**
Pul uninf 1/ Pul inf 1	69/ 179	527/667	11,57 %/ 21,15%	**0,55**
Pul uninf 2/ Pul inf 2	8/ 142	756/841	1,04% / 14,44%	**0,07**
Pul uninf 3/ Pul inf 3	66/ 174	393/ 674	14,37% / 20,52%	**0,70**
Gey uninf 2/ Gey inf 2	17/ 195	691/999	2,4% / 16,33%	**0,14**
Gey uninf 3/ Gey inf3	9/ 136	587/451	1,51% / 23,17%	**0,07**
Sample	Number of *Albugo* proteins	Number of *A. thaliana* proteins	*Albugo*/ total proteins	**Ratio uninf/inf**
Nc14 uninf 1/ Nc14 inf 1	12/ 162	921/880	1,29% / 15,54%	**0,08**
Nc14 uninf 2/ Nc14 inf 2	10/ 201	1352/1140	0,73% / 14,99%	**0,05**
Nc14 uninf 3/ Nc14 inf 3	19/ 342	849/1038	2,19% / 24,76%	**0,09**
Nc14 uninf 4/ Nc14 inf 4	28/ 232	885/1082	3,07% / 17,66%	**0,17**

Supplementary Table S2

Sample	Number of *Albugo* proteins	Number of *A. thaliana* proteins	*Albugo* / total proteins	**Ratio uninf/inf**
Ws0 field uninf 1/ Ws0 field inf 1.1	83/ 242	722/619	10,31 %/ 28,1%	**0,37**
Ws0 field uninf 1/ Ws0 field inf 1.2	83/ 283	722/757	10,31 %/ 27,21%	**0,38**
Sf2 field uninf 1	70	872	7,43%	
Sf2 field uninf 3	109	1024	9,62%	
Pul uninf/ Gey inf	109/277	939/1023	10,40% / 21,31 %	**0,49**
Gey uninf/ Gey inf	112/277	1044/1023	9,68% / 21,31 %	**0,45**
Sample	Number of *Albugo* proteins	Number of *A. thaliana* proteins	*Albugo*/ total proteins	**Ratio uninf/inf**
Ws0 lab uninf 1/ MPI1 inf 1	80/ 233	986/1142	7,50% / 16,95%	**0,44**
Ws0 lab uninf 2/ MPI1 inf 2	82/ 227	961/1126	7,86% / 16,77%	**0,47**
Ws0 lab uninf 3/ MPI1 inf 3	90/ 259	1033/1169	8,01% / 18,14%	**0,44**
Ws0 lab uninf 4/ MPI1 inf 4	108/ 329	1341/1489	7,45% / 18,10%	**0,41**

Supplementary Figure S2

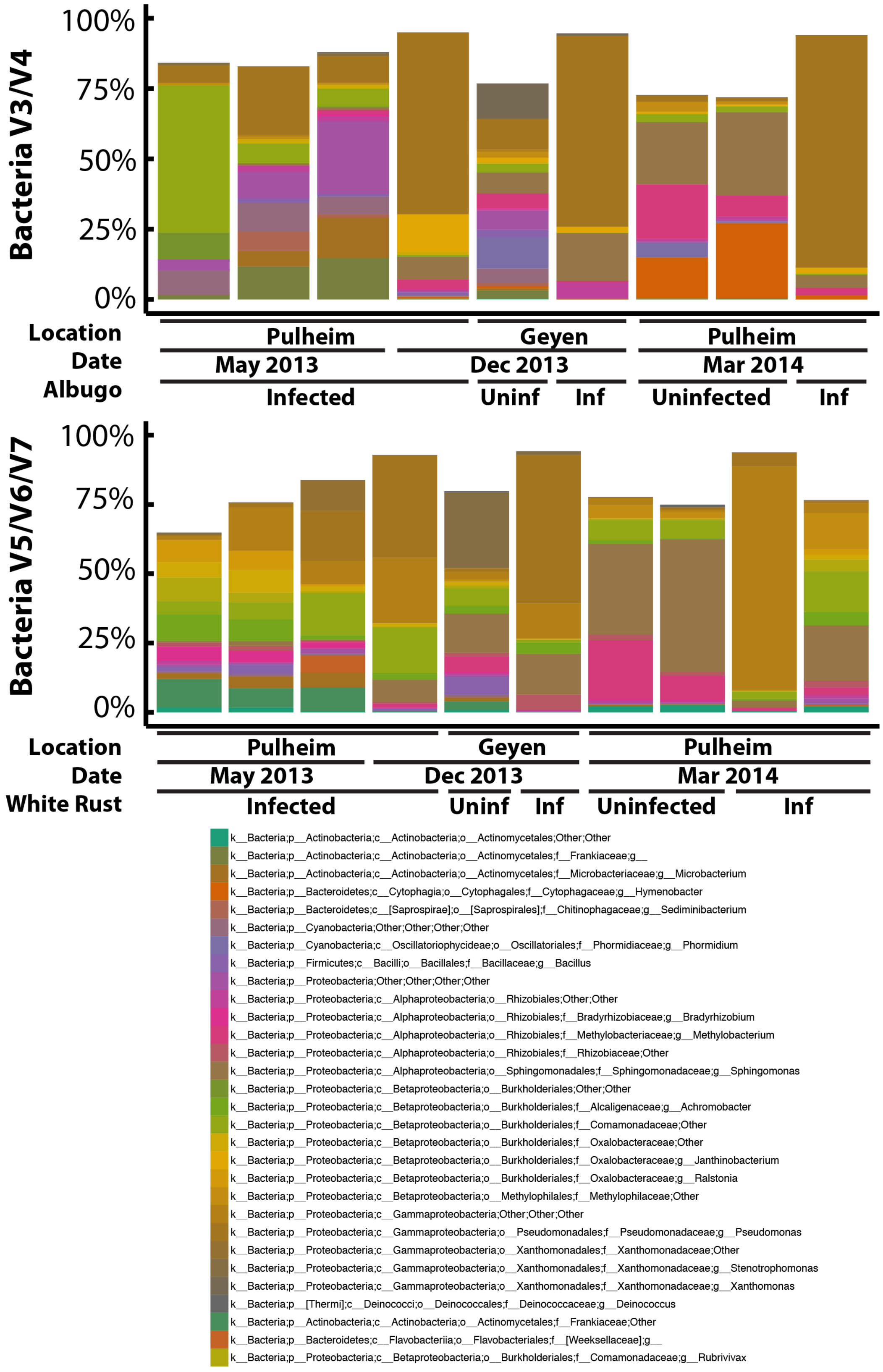

Supplementary Figure S3

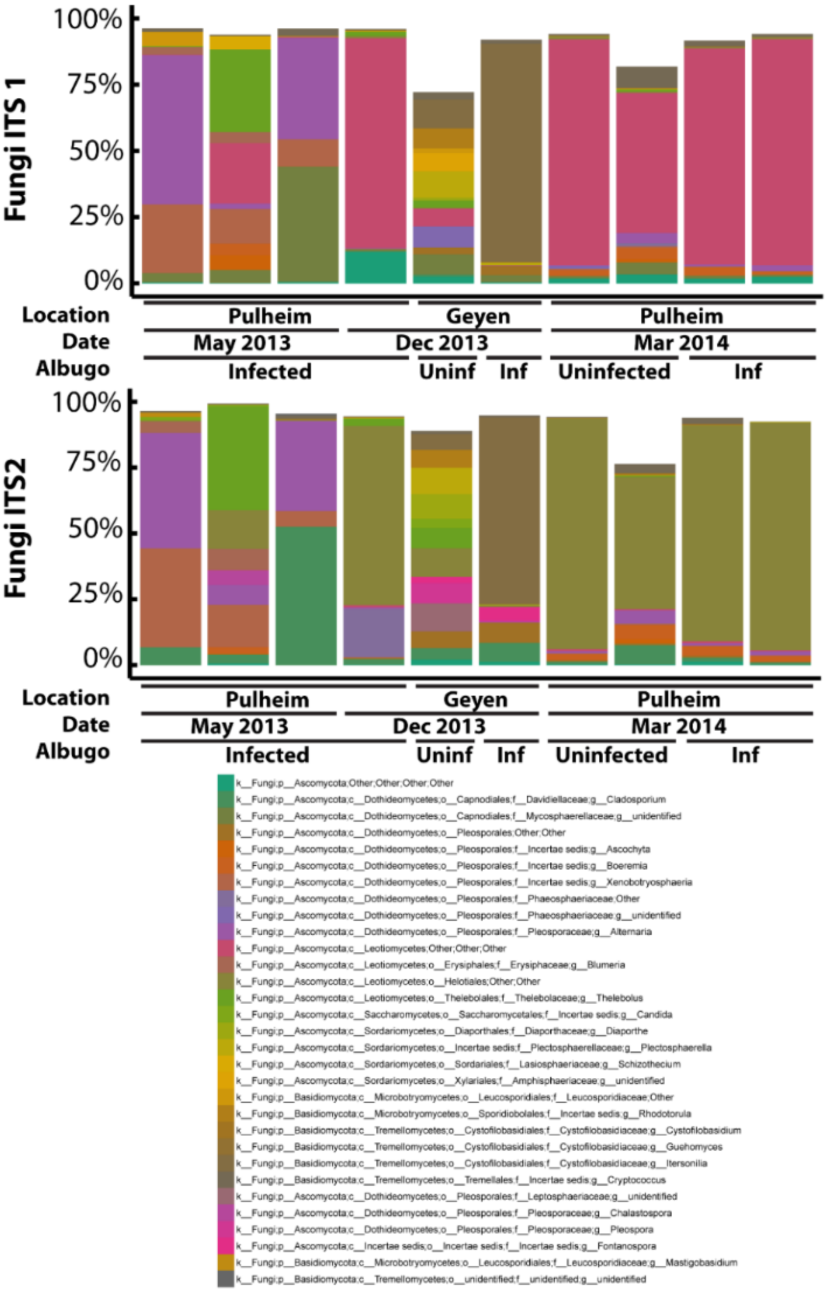

Supplementary Figure S4

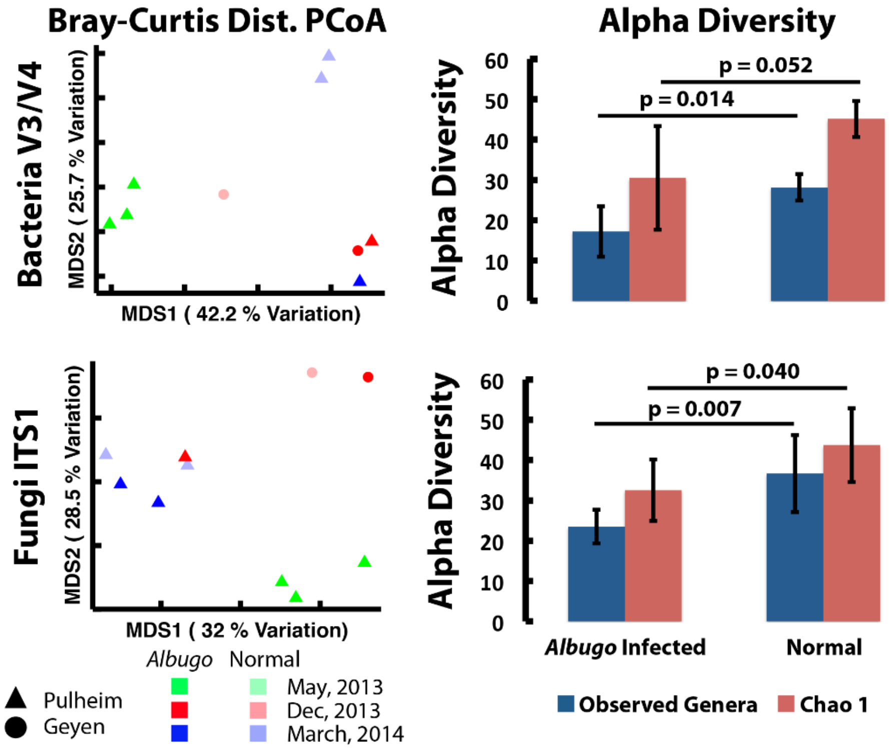

Supplementary Figure S5

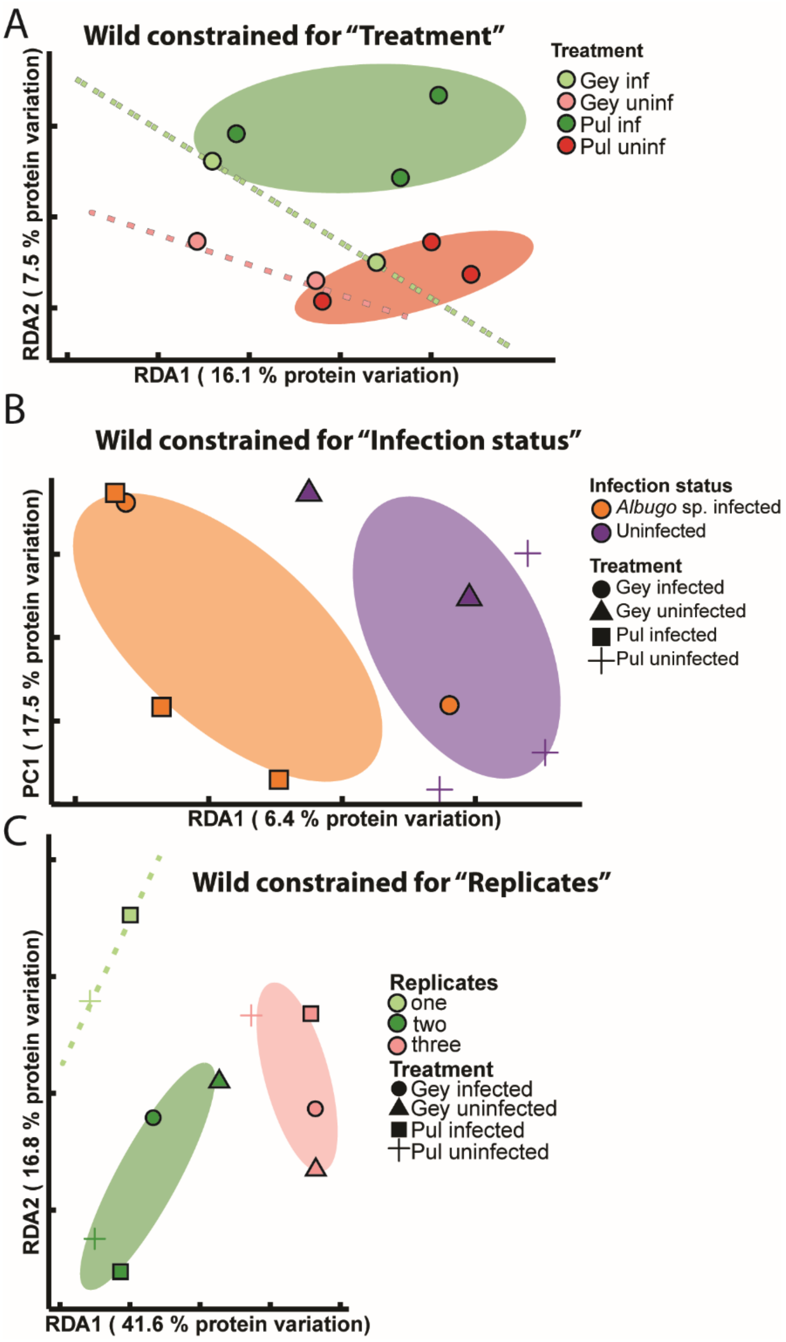

Supplementary Figure S6

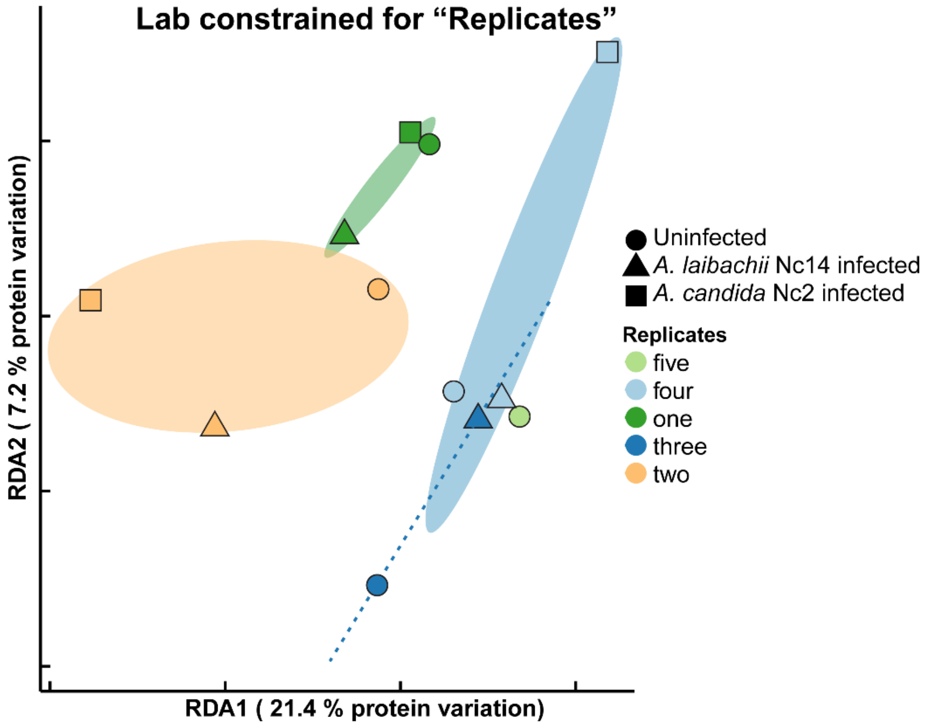

Supplementary Figure S7

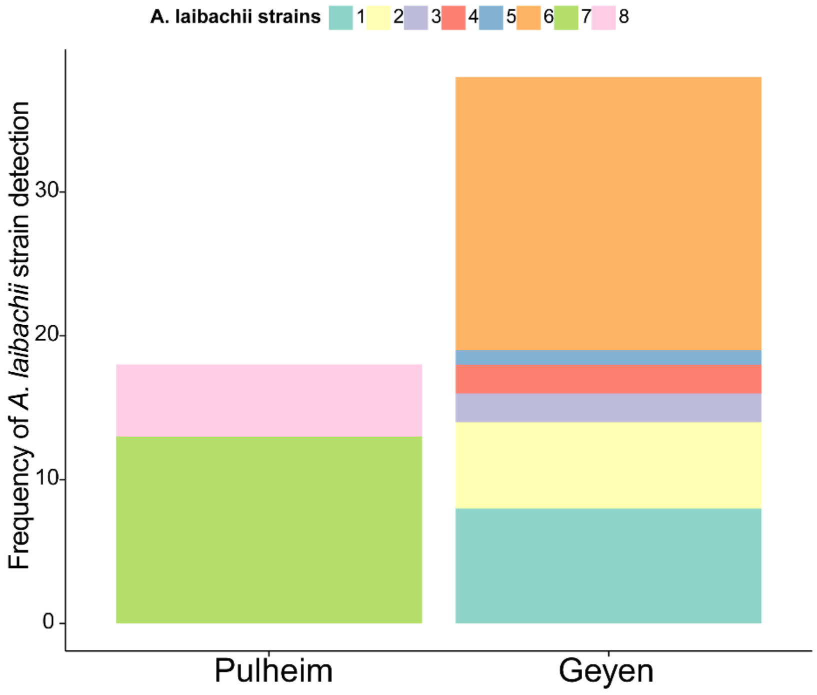

Supplementary Figure S8

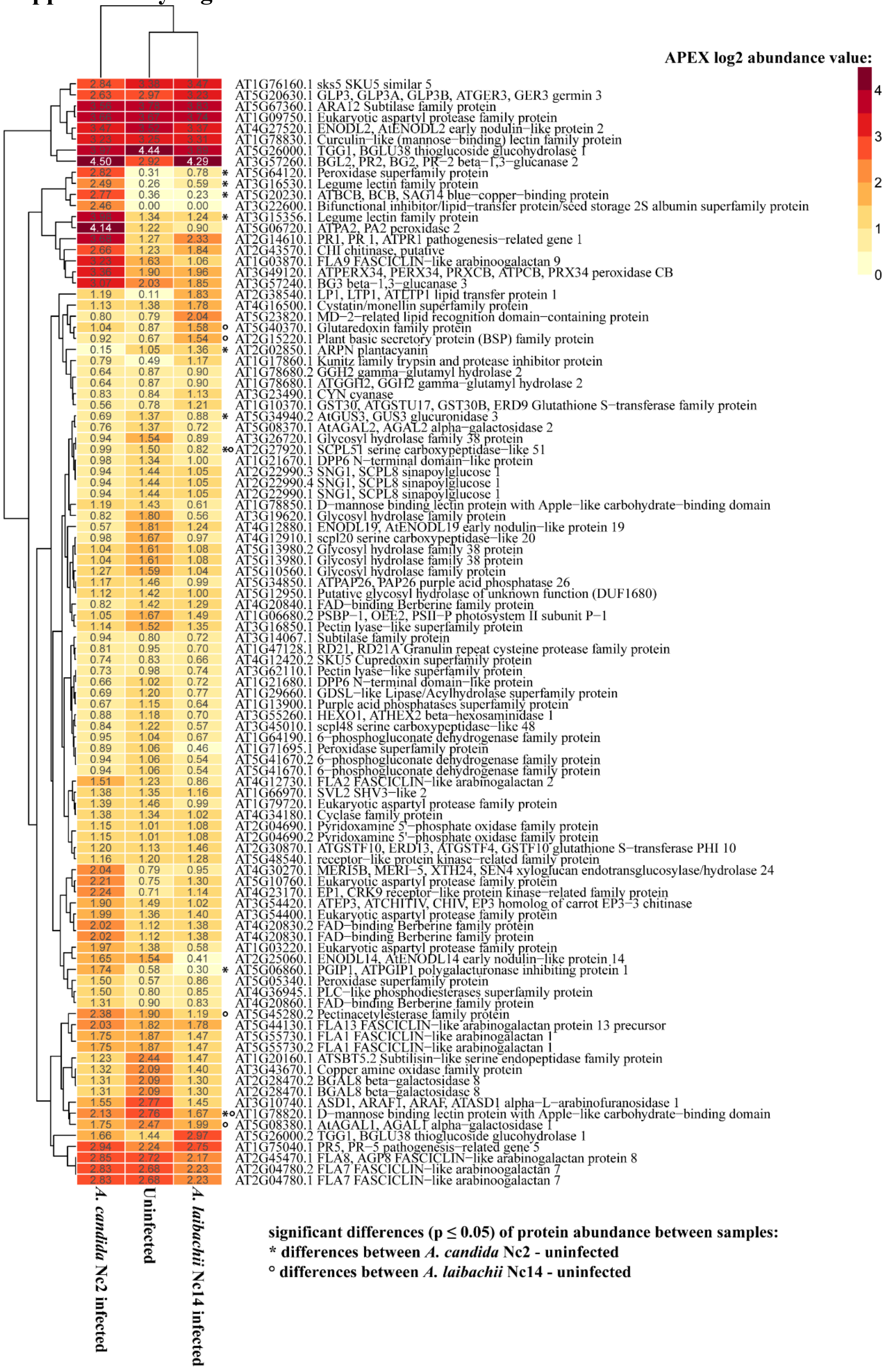

significant differences (p ≤ 0.05) of protein abundance between samples:
*** differences between *A. candida* Nc2 - uninfected**
° differences between *A. laibachii* Nc14 - uninfected
